# Mindfulness in primary care healthcare and teaching professionals and its relationship with stress at work: a multicentric cross-sectional study

**DOI:** 10.1186/s12875-021-01375-2

**Published:** 2021-02-02

**Authors:** Rosa Magallón-Botaya, Luis Angel Pérula-de Torres, Juan Carlos Verdes-Montenegro Atalaya, Celia Pérula-Jiménez, Norberto Lietor-Villajos, Cruz Bartolomé-Moreno, Javier Garcia-Campayo, Herminia Moreno-Martos, the Minduudd Collaborative Study Group, Luis Alberto Rodriguez, Ana Roldán-Villalobos, Elena Melús-Palazón, Francisco Javier Valverde, Nur Hachem-Salas, Leonor García-De Vinuesa, Carmen Morillo, Teresa Grande, Ronald Epstein, Luis Borau, Raquel Arias-Vega, Nur Hachem-Salas

**Affiliations:** 1 Family and Community Medicine Teaching Department of Zaragoza Sector 1, Institute of Health Carlos III (redIAPP 06/18), IIS-Aragon, Group B21-R17, University of Zaragoza, Zaragoza, Spain; 2 Multi-professional Teaching Unit for Family and Community Care of Córdoba, Healthcare District of Córdoba and Guadalquivir, Institute Maimónides of Research Córdoba (IMIBIC)/Hospital Reina Sofía/University of Córdoba, Isla de Lanzarote, 3, 14011 Córdoba, Spain; 3 Instittute of Health Carlos III (redIAPP 06/18), IIS-Aragon, Group B. University of Zaragoza, Zaragoza, Spain; 4 Family and Community Medicine Teaching Department of Burgos, Burgos, Spain; 5 Family and Community Medicine Teaching Department of Jaen, Jaen, Spain; 6 Hospital Universitario Miguel Servet, Zaragoza, Spain; 7 Multi-professional Teaching Unit for Family and Community Care of Almeria, Almeria, Spain

**Keywords:** Mindfulness, Work stress, Subjective well-being, Primary health care professionals, Health services research

## Abstract

**Background:**

Work stress is a common problem among the health personnel of the Spanish National Health System. The objective of this paper is to assess the state of mindfulness among Spanish primary care providers and to evaluate its potential relationship with work stress and basic labor and sociodemographic characteristics.

**Methods:**

Cross-sectional, multi-centric study. Primary care nurses, teachers, teaching collaborators and residents assigned to six Spanish Family Medicine/Family and Community Care Departments were invited to participate (*n* = 475). A template was designed in Google Forms, including sociodemographic and work-related variables. The state of mindfulness was measured with the Five Facet Mindfulness Questionnaire (FFMQ), while work-related stress was measured using an ordinal scale ranging from 0 to 10 points. Descriptive and inferential statistical analyses were carried out, as well as bivariate and multivariate statistics.

**Results:**

The mean age of participants was 40,14 ± 13.12 (range:23–65 years); 66.9% were women, 42.5% internal medicine residents, 29.3% family physicians, and 20.2% nurses. More than half (54.5%) knew about mindfulness, with 24.0% have received training on it, and 22.5% were usual practitioners. The average level of mindfulness was 127.18 ± 15.45 (range: 89–177). The average score of stress at work was 6.00 ± 2.44; 49.9% (range: 0–10). 49.9% of participants scored 7 or more on the stress at work scale. There was an inverse correlation between the levels of mindfulness (FFMQ total score) and work-related stress (Spearman’s *r* = − 0.155, *p* = 0.003). Significant relationships between the mindfulness practice and the level of mindfulness (F = 29.80, *p* < 0.001), as well as between the mindfulness practice and the level of work-related stress (F = 9.68, *p* = 0.042), were also found.

**Conclusions:**

Levels of mindfulness in primary care health providers were in line with those levels observed in other groups of health professionals. Half of all of the primary care providers suffered from a high degree of stress. Although weak, inverse relationships were observed between levels of mindfulness and stress at work, with lower values of stress at work among those who practiced mindfulness.

**Trial registration:**

NCT03629457.

**Supplementary Information:**

The online version contains supplementary material available at 10.1186/s12875-021-01375-2.

## Background

A great number of healthcare professionals complain about their work conditions: excess of health care pressure, increasingly demanding users seeking treatment for their health problems and needs, a lack of sufficient time for training and re-training, as well as the perceived lack of support by managers and their superiors [1–3]. Professionals who also take on training tasks, suffer situations of even more overload. Many studies have suggested the need to solve this with an integral form of tackling the physical and psychological consequences of work stress in healthcare professionals. Not merely adopting measures at a work organization level and their work conditions, but also providing individual tools to handle stress, based on acceptance of reality and emotional self-regulation [4, 5].

In the specific area of post-graduate teaching, Spain boasts a high-quality model of specialized training [6]. The Training Program for Resident Intern Specialists (RIS) from the Family and Community Medicine specialty is 4 years long and the program for Family and Community Nursing is 2 years. Although the goal is for the young training doctor or nurse, to take on responsibilities in different areas of competence, both healthcare, teaching and research, in a progressive manner, stress is inevitable, especially in the emergency department [7]. An important figure in the training process of the RIS is the personal tutor. Carrying out this task of teaching implies a major work load in addition to the regular everyday work activity.

The term “mindfulness” originates in Buddhist teachings and implies a state, in which the individual focuses their present on the task at hand, without the mind digressing to the future or past, and without the sensation of attachment or rejection. Mindfulness is a quality that can be developed through appropriate training [8] and that generates significant benefits in the physical and mental health of those engaging in it. The level of mindfulness appears to be an influential factor in the determination of the degree of work-related stress experienced by healthcare professionals. In fact, diverse intervention studies and meta-analyses carried out on healthcare professionals, have demonstrated that meditation techniques increase the levels of mindfulness and improve coping skills for work-related stress [9, 10].

We have barely found studies conducted in our field that examine the state of mindfulness in primary care healthcare professionals, on the one hand, with the potential relationship between the knowledge, training and the practice of mindfulness; and on the other, with the level of work-related stress perceived [9, 11]. Furthermore, we are unaware of its relationship with the teaching and learning condition of the professional. Therefore, this study aims to determine the state of mindfulness between Spanish primary care professionals, as well as to assess potential associations between the levels of mindfulness, the practice of mindfulness exercises and the degree of work-related stress. All this focused, especially, to the teaching areas, comparing the results according to the primary care professional profile.

## Methods

### Design

A cross-sectional study was conducted, using a self-administered online questionnaire, by healthcare professionals in the primary care teaching and care environment.

### Participants and procedure

The inclusion criteria were: 1) To be a family doctor, a family medicine resident, or a primary care nurse; and 2) To provide informed consent for participation in the study. The study population consisted of primary care professionals from six Teaching Departments of the Spanish National Health System of different dimensions, according to the population density of each territory, distributed across the Spanish geography, (N total = 802; Córdoba *N* = 256; Almería, *N* = 147; Jaén, *N* = 185; Burgos, *N* = 64; Ponferrada, *N* = 63; and Zaragoza, *N* = 87). Of the surveyed participants professionals, 297 were teachers and 595 were Resident Intern Specialists in Family and Community Medicine/Nursing. For comparison purposes, a subsample of 267 nurses working in non-teaching care settings, in the Córdoba province, were also surveyed. The results from this sample have already been published [12]. Based on the results of previous research [12], it was necessary to recruit at least 433 participants in the present study. The sample size was calculated assuming a precision of ±3.5%, a confidence of 95%, and an expected knowledge about mindfulness of 36.5%. The calculations have been made with the GRANMO program (http://www.imim.cat/ofertadeserveis/software-public/granmo/). For the recruitment of the subjects, the study was disseminated by sending an email to all those responsible for the participating centers.

### Measurements

The participation procedure was carried out electronically, through an online questionnaire created by our research group in Google Forms. The questionnaire was originally tested on a subsample of four subjects, in order to check its comprehensibility. The questionnaires were anonymous and the appropriate mechanisms were established to safeguard the confidentiality of the information collected.

Mindfulness was the main variable and it was measured using the Five Facet Mindfulness Questionnaire (FFMQ), validated for the Spanish population [13]. The FFMQ consists of 39 items and five dimensions: observation, description, acting conscientiously, lack of judgment and lack of reactivity. The participants answered to each item on a Likert scale, with response items ranging from 1 (never or rarely true) to 5 (very often or almost true). The FFMQ permits the computation of a total mindfulness score, from the total of all of its dimensions, such that higher scores mean greater levels of mindfulness. The psychometric characteristics of the FFMQ in the Spanish population are appropriate, with a response range from between 89 and 177. Computation of internal consistency for the present data showed an alpha coefficient of 0.88 for the FFMQ total score. In the correlations with other scales, the FFMQ validated for the Spanish population showed an expected and significant relationship with almost every scale, except the observation dimension [13]. In addition to the state of mindfulness, which was measured by the FFQM, other variables were also included, such as age, gender, professional profile, work site, labor seniority, degree of awareness of the mindfulness concept, prior training, level of practice and frequency. The level of work-based stress was also assessed for the prior 2 weeks, using a visual analogue ordinal scale. Participants were asked to rate their work-based stress using the following question: On a scale from 0 (none) to 10 (maximum), what degree of work stress have you had in the last 2 weeks at your usual job?. In previous studies, this type of scale has been found to be suitable for the assessment of perceived stress [14].

### Ethical considerations

The study protocol was approved by the Ethics and Clinical Research Committee of Córdoba, (number 275; referencie 3845). Information on the study’s objective was provided online to the professionals who agreed to participate via informed consent prior to completing the questionnaire. In accordance with personal data protection and confidentiality regulations (European Regulation on data protection and in accordance with Organic Law 3/2018 on Personal Data Protection and Digital Rights Guarantee).

### Statistical analysis

Data centralization, dispersion and position variables were used, as well as absolute and relative frequencies to describe the variables that were the subject of the study, based on their distribution. Confidence intervals were calculated at 95% (95% CI) for the main estimators. In order to examine the relationship between the sociodemographic and labor variables, and the degree of mindfulness or the level of work-related stress, bivariate analyses were conducted, applying the Spearman’s correlation coefficient (r), the Student’s t test and the ANOVA test (upon verifying the normality of the variables through the Kolmogorov-Smirnov test; in the contrary case, either the Spearman’s correlation coefficient, Mann-Whitney U test or the Kruskal-Wallis test was used). Multivariate analyses were also applied, through application of multiple linear regression models, using the dependent variables of mindfulness and level of work-related stress and the independent variables of age, gender, profession, amount of time worked and knowledge, training and practice of mindfulness, in model 1 and of these only those whose *p* value was less than 0.05 were considered in model 2. The statistical significance level was established at alpha error ≤ 0.05. The SPSS (Statistical Package for the Social Sciences) v.22 was used for data analysis. SPSS is a statistical computer program widely used in the social and applied sciences (https://www.ibm.com/products/spss-statistics).

## Results

The survey was completed by 475 primary care health workers, with an overall response rate of 44.5%. The response rate varied between 47.2% of the members of the teaching units and 35.9% of the non-teaching nurses. The mean age was 40.14 ± 13.12 –SD- (range: 23–65 years). 66.9% of the respondents were women. 42.5% were resident intern physicians; 29.3% were family doctors and 20.2% were nurses. Of the physicians, 61.0% had worked for over 20 years, as was the case with 42.7% of the nurses, whereas 91.2% of the resident intern physicians had been working for less than 4 years.

The mean score for work-related stress was 6.00 ± 2.44 (range: 0–10, 95% CI: 5.78–6.22), with 49.9% of the participants scoring 7 or higher on the work-related stress scale.

Regarding mindfulness, 54.5% stated that they knew the meaning of this concept; 24.0% of them, had received prior training on its practice, while 22.5% currently engaged in mindfulness exercises. Of this 22.5, 4% of the surveyed participants practiced mindfulness daily, while 4.2% practiced it from two to three times a week and 14.3% did so occasionally.

The mean level of mindfulness, assessed with the FFMQ questionnaire, was 127.18 ± 15.45 (range: 89–177; 95% CI: 125.79–128.57).

As seen in Table 1, using bivariate analysis, it was possible to observe statistically significant differences between the levels of mindfulness and age (*p* < 0.001; highest mean score on the FFMQ for the oldest professionals), professional category (*p* < 0.001; lowest score for the resident intern physicians), amount of time worked (*p* < 0.001; higher mean score for professionals having worked for the longest amount of time), and training or prior practicing of mindfulness (*p* < 0.001). No significant differences were found based on gender (*p* = 0.910) or prior knowledge of the concept of mindfulness (*p* = 0.145).

**Table 1:** Relationship between the study variables and the state of mindfulness through a bivariate analysis

Variables	Mean ± SD (95% CI of the mean)	*P* value
Age (years):		< 0.001*
-Under 30	121.94 ± 13.49 (119.95–123.90)	
-From 30 to 45	129.75 ± 14.93 (126.81–132.70)	
-46 or over	130.81 ± 16.14 (128.51–133.10)	
Gender:		0.910**
-Male	127.29 ± 14.68 (124.98–129.61)	
-Female	127.12 ± 15.84 (125.37–128.87)	
Professional category:		< 0.001*
-Family physician	130.62 ± 15.29 (128.31–132.92)	
-Nurse	131.19 ± 16.39 (127.88–134.49)	
- Internal medicine residents	122.42 ± 13.83 (120.52–124.32)	
Amount of time worked:		< 0.001*
-Less than 4 years	122.59 ± 13.75 (120.70–124.48)	
-From 5 to 10 years	130.59 ± 16.76 (125.78–135.40)	
-From 11 to 20 years	127.35 ± 15.83 (123.63–131.07)	
-Over 20 years	132.36 ± 15.45 (129.88–134.84)	
Aware of mindfulness:		0.145**
-Yes	128.12 ± 15.87 (126.18–130.07)	
-No	126.05 ± 15.45 (124.05–128.04)	
Has received training on mindfulness:		< 0.001**
-Si	130.73 ± 18.01 (127.39–134.07)	
-No	126.06 ± 14.40 (124.57–127.55)	
Practices mindfulness:		< 0.001*
-Every day	145.53 ± 17.85 (136.92–154.13)	
-From 2 to 3 times/week	136.00 ± 15.82 (128.59–143.41)	
-Occasionally	129.35 ± 15.82 (123.87–126.83)	
-Never	125.35 ± 14.43 (123.87–126.83)	

*SD* Standard deviation, *95% CI* 95% confidence interval

* ANOVA test; ** Student’s t test

Table 2 shows a statistically significant association between the stress-related work situation of the professional and his/her age (*p* < 0.001), professional profile (*p* < 0.001), amount of time worked (*p* < 0.001), and the practice of mindfulness (*p* < 0.001). In the bivariate analysis shown in this table, no significant differences were found, with regards to gender (*p* = 0.714), knowledge (*p* = 0.727) or prior training in mindfulness (*p* = 0.251).

**Table 2:** Relationship between the study variables and the level of work-related stress through a bivariate analysis

Variables	Mean ± SD (95% CI of the mean)	*P* value
Age (years):		< 0.001*
-Under 30	5.38 ± 2.61 (5.00–5.77)	
-From 30 to 45	6.36 ± 2.40 (5.88–6.83)	
-46 or over	6.39 ± 2.18 (6.08–6.70)	
Gender:		0.714**
-Male	6.10 ± 2.31 (5.76–6.46)	
-Female	5.95 ± 2.50 (5.67–6.22)	
Professional category:		< 0.001*
-Family physician	6.34 ± 2.03 (6.04–6.65)	
-Nurse	6.51 ± 2.39 (6.02–6.99)	
- Internal medicine residents	5.47 ± 2.68 (5.10–5.83)	
Amount of time worked:		< 0.001*
-Less than 4 years	5.40 ± 2.66 (5.03–5.76)	
-From 5 to 10 years	6.22 ± 2.08 (5.63–6.82)	
-From 11 to 20 years	6.72 ± 2.13 (6.22–7.22)	
-More than 20 years	6.40 ± 2.20 (6.04–6.76)	
Aware of mindfulness:		0.727**
-Yes	6.02 ± 2.67 (5.75–6.30)	
-No	5.96 ± 2.64 (5.61–6.32)	
Has received training on mindfulness:		0.251**
-Yes	5.85 ± 2.37 (5.41–6.29)	
-No	6.04 ± 2.47 (5.79–6.30)	
Practices mindfulness:		0.040*
-Every day	4.58 ± 2.34 (3.45–5.71)	
-From 2 to 3 times/week	6.70 ± 1.84 (5.84–7.56)	
-Occasionally	5.96 ± 2.39 (5.38–6.53)	
-Never	6.04 ± 2.47 (5.79–6.29)	

*SD* Standard deviation, *95% CI* 95% confidence interval

* Kruskal-Wallis Test; ** Mann-Whitney U

A direct, statistically significant relationship was found between the degree of exercise practice and level of mindfulness, assessed in the FFMQ questionnaire (F = 14.389, *p* < 0.001). A slight, but significant negative correlation was also found between the level of mindfulness (FFMQ overall score) and the level of work-related stress (*r* = − 0.155, *p* = 0.003).

In the multivariate analysis (Table 3), variables associated with the level of mindfulness were: age (B = 0.313; *p* < 0.001), practice of mindfulness (B = 4.614; *p* < 0.001), and work-related stress (B = − 1.044; *p* < 0.001). On the other hand, as seen in Table 4, levels of work-related stress are associated with age (B = 0.036; *p* < 0.001) and level of practicing of mindfulness (B = -0.334; *p* < 0.001). In contrast with the findings from the bivariate analysis, as seen in Tables 3 and 4, the multivariate analysis did not reveal significant relationships, between professional profile and levels of mindfulness, or perceived work-related stress.

**Table 3:** Variables associated with the state of mindfulness through a multivariate analysis

Variables	Model 1				Model 2			
	B	95.0% CI for B		p	B	95.0% CI for B		
		Lower Bound	Upper Bound			Lower Bound	Upper Bound	p
Age	0.202	−0.015	0.419	0.068	0.313	0.211	0.414	< 0.001
Gender	0.533	−2.276	3.343	0.709	–	–	–	–
Profession	−1.071	−3.626	1.485	0.411	–	–	–	–
Time working in current position	0.286	−0.681	1.254	0.561	–	–	–	–
Awareness of mindfulness	1.039	−1.970	4.048	0.498	–	–	–	–
Training in mindfulness	−0.463	−4.047	3.122	0.,800	–	–	–	–
Mindfulness practice	4.727	2.720	6.734	< 0.001	4.614	2.845	6.383	< 0.001
Work-related stress	−1.062	− 1.603	− 0.520	< 0.001	− 1.044	−1.579	− 0.508	< 0.001
Intercept	118.461	–	–	–	114.675	–	–	–

Maximum model (model 1). Parsimonious model (model 2). Dependent variable: mindfulness (FFMQ); Coefficient of determination of model 1: R^2^ = 0.163 (F = 11.35; *p* < 0.001); Coefficient of determination of model 2: R^2^ = 0.161 (F = 29.80; *p* < 0.001). *n* = 474; *95% CI* 95% confidence interval

**Table 4:** Variables associated with the state of work-related stress through a multivariate analysis

Variables	Model 1				Model 2			
	B	95.0% CI for B		p	B	95.0% CI for B		
		Lower Bound	Upper Bound			Lower Bound	Upper Bound	p
Age	0.000	−0.037	0.036	0.976	0.036	0.019	0.052	< 0.001
Gender	0.036	−0.436	0.507	0.882	–	–	–	–
Profession	−0.035	− 0.464	0.394	0.871	–	–	–	–
Time working in current position	0.186	0.024	0.347	0.024	–	–	–	–
Awareness of mindfulness	−0.318	− 0.822	0.187	0.216	–	–	–	–
Training in mindfulness	0.241	−0.360	0.843	0.431	–	–	–	–
Mindfulness practice	−0.378	− 0.714	−0.043	0.027	−0.334	− 0.631	−0.037	< 0.001
Intercept	5.771	–	–	–	5.015	–	–	–

Maximum model (model 1). Parsimonious model (model 2). Dependent variable: perceived work-based stress; Coefficient of determination of model 1: R^2^ = 0.054 (F = 8.86; *p* = 0.002); Coefficient of determination of model 2: R^2^ = 0.039 (F = 9.68; *p* < 0.001). *n* = 474; *95% CI* 95% confidence interval

## Discussion

Half of the healthcare professionals appear to suffer from a high degree of work-related stress. There is a positive, though weak, correlation between the degree of mindfulness and work stress, with less stress among those who practice mindfulness. In this sense, the state of mindfulness in primary care healthcare providers appears to be in line with that observed in other professional healthcare groups [15], with a level of knowledge more than acceptable, although much lower in terms of practice.

This finding supports the potential protective role of mindfulness in the reduction of work-related stress. Meditative practice might be associated with greater levels of mindfulness, and this, in turn, to the reduction of perceived work -related stress. Some prior studies are in line with this explanatory model [16, 17].

The multivariate analysis demonstrates that there are no differences between the different professional profiles that were analyzed: physicians, nurses and residents in training, both with respect to the level of mindfulness as well as the level of work-related stress. These variables have been studied separately for the distinct types of professionals [18]. This study brings the novelty of comparing at the same time the level of mindfulness and stress between different professional groups. There appears to be a discrepancy in this result, since a priori it appears that stress may be more closely related to the level of professional responsibility.

It has been sufficiently accredited that emotional intelligence prevents burnout and work-related stress, not only in physicians but also in nurses [19, 20]. And the practice of mindfulness is another strategy used for emotional reinforcement. It is a technique which, when put into practice, eliminates (in theory) the anticipatory anxiety, which is a trigger for work-related stress. Some studies carried out in primary health care professionals, show that meditation techniques serve to improve coping with stress and empathy [16]. It is likely that the effectiveness of these programs, in preventing work-related stress is closely related to the capacity for self-compassion [21]. An attitude of self-compassion towards oneself, is one of the key elements of mindfulness, since it permits us, to better manage difficult emotions, such as fear, anger, sadness or doubt.

Our multidisciplinary research group has also examined the specific effect of mindfulness on nursing [12] and other primary care professionals, as we have detailed previously [21].

In the study carried out among the nursing staff [12] it was found that those nurses who practiced mindfulness had lower levels of work stress than those who did not. Within the dimensions of the FFMQ, they showed a greater capacity for observation, in addition to a higher level of global mindfulness, compared to other primary care health professionals, even higher than in those who received a training program in mindfulness [21].

Recently, Aranda et al. [22] evaluated the effectiveness of an eight-week training program on mindfulness and self-compassion, to reduce stress levels and burnout in primary care professionals. Despite the limited response to the program, it suggests the potential benefits of promoting mindfulness and practices of self-compassion in the healthcare environment.

Other interesting experiences are available, such as the program proposed approximately one decade ago, by Krasner, Epstein et al. [23], with primary care physicians. The results were conclusive: the participation in a communication program, was associated with short and long-term improvements, in well-being and attitudes associated with patient-centered care.

Delving into the education field, it is especially interesting the fact observed by Beddoe et al. [24] that «being attentive» not only decreases personal stress in students of a training intervention, but also improved their empathetic attitude and decreased the tendency to be loaded down with negative emotions of others.

Also, in the teaching environment, certain experiences in the area of effectiveness of mindfulness in education have had positive results, not only for teachers [25].but also for students [26], with substantial improvement taking place in academic performance.

There is a peculiarity of our study that makes it different from others. We are in a special teaching and learning environment: healthcare. Teachers and residents share expectations and responsibilities of teaching and learning with the care activity and the stress that may be caused by this situation. The work of the teacher tutors of the Family Medicine Departments, does not differ substantially from that of the purely educational environment. But perhaps the complementarity of their teaching competencies, with the clinical practice, makes the latter considered a priority undermining the teaching activity.

The effectiveness of mindfulness techniques on the professionals (such as physicians and nursing residents) in the learning process, has been demonstrated in the past [27] as well as recently [28]. However, it does not appear that these evidences had been accompanied by its serious and ongoing implementation to reduce stress in the areas that have been studied.

In our study, we analyze the prior level of mindfulness and its potential relationship with some variables. We have not found differences in mindfulness nor in the level of work-related stress, by professional groups: in nurses, physicians and residents, suggesting that intervention programs may be useful for all of the professional groups.

There are no differences detected in terms of gender, but there were, with regards to age. In this sense, one of the pending areas to be addressed, also suggested in other studies, is the lack of assessment of the long-term effectiveness of the intervention programs.

An inverse relationship was observed between the level of practicing of mindfulness exercises and the level of work-related stress, such that, with greater practice levels, there were lower work-related stress levels. This coincides with the results from past studies and supports the need for the practicing of mindfulness in the care and training environments.

Limitations include the sample composition (unless it can be demonstrated that it maintains the same percentages by occupation as the general Spanish population). It should be mentioned that at least the sample size is high, taking into account the type of statistical analysis carried out, therefore the statistical power was adequate. Another limitation is the use of a scale with only one item to assess perceived work-related stress, since this is a complex construct. However, this work is an exploration that should be confirmed in future studies. The final limitation is the type of design used, which, being cross-sectional, does not permit us to establish causal relationships. Cross-sectional data is always limited to examine a research question which entails a relationship (mindfulness reduces stress). But maybe it is the other way round and those people who have less stress, have more time to do practice mindfulness. So, in future studies, the potential causal relationships between variables derived from the observed association should be studied using appropriate designs.

## Conclusions

Given the high percentage of primary care providers who experience work-related stress, and the fact that Mindfulness-Based Interventions seem to be useful to reduce this problem, health authorities and managers in charge of continuous training programs should take them into account in the primary care setting.

Mindfulness-Based Interventions are very versatile techniques, ideal for primary care contexts or similar, and that achieve their greatest degrees of effectiveness not only in patients with symptoms of stress, anxiety or depression, but also in health professionals. It is necessary to carry out intervention studies directed at these health professionals in order to verify if the standardized programs of instruction in mindfulness manage to reduce the level of stress and burnout.

## Supplementary Information

**Additional file 1.**

## Data Availability

The raw data supporting our findings is in the companion file (Supplementary Material).

## Abbreviations

FFMQ: Five Facet Mindfulness Questionnaire
RIS: Resident Intern Specialists
SD: Standard deviation
95% CI: 95% confidence interval
SPSS: Statistical Package for the Social Sciences
